# Perfusion Changes at the Forehead Measured by Photoplethysmography during a Head-Down Tilt Protocol

**DOI:** 10.3390/bios9020071

**Published:** 2019-05-27

**Authors:** Tomas Ysehak Abay, Kamran Shafqat, Panayiotis A. Kyriacou

**Affiliations:** Research Centre for Biomedical Engineering, City, University of London, London EC1V 0HB, UK; kamran79s@hotmail.com (K.S.); p.kyriacou@city.ac.uk (P.A.K.)

**Keywords:** reflectance photoplethysmography, haemoglobin concentrations, Near Infrared Spectroscopy, head-down tilt, venous pooling

## Abstract

Photoplethysmography (PPG) signals from the forehead can be used in pulse oximetry as they are less affected by vasoconstriction compared to fingers. However, the increase in venous blood caused by the positioning of the patient can deteriorate the signals and cause erroneous estimations of the arterial oxygen saturation. To date, there is no method to measure this venous presence under the PPG sensor. This study investigates the feasibility of using PPG signals from the forehead in an effort to estimate relative changes in haemoglobin concentrations that could reveal these posture-induced changes. Two identical reflectance PPG sensors were placed on two different positions on the forehead (above the eyebrow and on top of a large vein) in 16 healthy volunteers during a head-down tilt protocol. Relative changes in oxygenated (ΔHbO${}_{2}$), reduced (ΔHHb) and total (ΔtHb) haemoglobin were estimated from the PPG signals and the trends were compared with reference Near Infrared Spectroscopy (NIRS) measurements. Also, the signals from the two PPG sensors were analysed in order to reveal any difference due to the positioning of the sensor. ΔHbO${}_{2}$, ΔHHb and ΔtHb estimated from the forehead PPGs trended well with the same parameters from the reference NIRS. However, placing the sensor over a large vasculature reduces trending against NIRS, introduces biases as well as increases the variability of the changes in ΔHHb. Forehead PPG signals can be used to measure perfusion changes to reveal venous pooling induced by the positioning of the subject. Placing the sensor above the eyebrow and away from large vasculature avoids biases and large variability in the measurements.

## 1. Introduction

The forehead is one of the common locations for measuring arterial oxygen saturation (SpO${}_{2}$) by pulse oximetry. To estimate the SpO${}_{2}$, pulse oximeters rely on photoplethysmography (PPG), which is an optoelectronic technique that measures changes in blood volume in the microvasculature [1]. The forehead region is well perfused by arteries branching from the internal carotid, thus, providing good quality and reliable PPG signals [2,3,4]. PPG measurements acquired from the forehead have been reported to be less affected by vasoconstriction and to be quicker than fingers in indicating deoxygenations [2,3,5,6]. In addition, the forehead is less susceptible to movements and, for intra-operative monitoring, it is a more accessible location than fingers [4]. Forehead PPG can also be used to monitor other physiological parameters such as heart rate [7] or as a replacement of pulse pressure variations in the arterial blood pressure [8].

However, the PPG signals acquired from the forehead contain a stronger venous component compared to the finger [3,9]. In certain situations, this venous component can be worsened by the positioning of the patients such as in the Trendelenburg position [3,4,10]. The venous presence in the PPG measured at the forehead can usually be identified in the form of cardio-synchronous venous pulsations, which deteriorate the quality of the signal [3]. In the presence of these cardio-synchronous venous pulsations, pulse oximetry readings acquired from the forehead could be lower than those measured at the fingers because of the mixture of venous and arterial blood in the measurement area [4,10,11].

To overcome these limitations, the application of a low pressure on the sensor was suggested for pushing venous blood from beneath the sensor [3,4,10]. This low pressure aims at removing the venous blood (pulsations) from the PPG signals by mechanically collapsing the venous circulation in the measurement area. This approach allows reducing the venous contribution to the signal without affecting the arterial component, thus providing more reliable SpO${}_{2}$ readings [3,4].

To mechanically suppress the venous component from forehead PPG measurements, pulse oximeters manufacturers provide a headband along with their forehead sensors. Although efficient most of the times, these headbands do not guarantee the full solution to the problem. Since there is no standard measure of the actual pressure applied, the headband may suppress arterial pulses (in particular in hypotensive patients) by exerting an excessive pressure and it can cause pressure-induced injuries in the measuring site [3]. In addition, the headband can slip-off from the patient during the measurement and the applied pressure may not be sufficient to suppress the venous component in the signal [12]. To date, there is no method to measure the presence of venous blood underneath the sensor. Venous pulsations can only be visually recognised in the PPG signals, or the venous presence can be presumed from the position of the patient (e.g., Trendelenburg).

Another factor that may influence PPG/SpO${}_{2}$ readings at the forehead is the presence of large blood vessels. This anatomical location is populated with large arteries and veins lying between the skin and scalp. The presence of these large vessels can cause errors in the measurements, increasing the unreliability of the measurements [2].

In previous publications, we introduced the possibility of estimating changes in concentrations of oxygenated, reduced, and total haemoglobin from dual-wavelength PPG signals acquired at the forearm. The estimated parameters demonstrated to be able to indicate changes in perfusion in the measurement area during vascular occlusions on the arm [13,14]. In this study, we investigate whether PPG signals acquired from the forehead could be used to estimate relative changes in oxygenated, reduced, and total haemoglobin at the forehead during a gravitational change (i.e., negative bed tilting). Estimating haemoglobin concentrations, in particular, reduced haemoglobin, from the same PPG/SpO${}_{2}$ sensor on the forehead can provide an indication of the increased (or decreased) presence of venous blood underneath the sensor (i.e., venous blood pooling). As a validation, the changes in the estimated parameters were compared with state-of-the-art Near Infrared Spectroscopy (NIRS) monitoring. In addition, the effect that a large vein on the forehead may have in the quantification of changes in haemoglobin concentrations is investigated by comparing two identical PPG sensors placed on two different locations on the forehead.

## 2. Methods

### 2.1. Measurement Instrumentation

**Figure 1:**
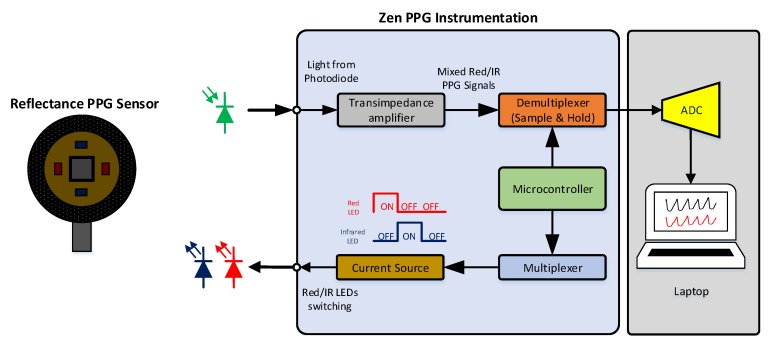
Block diagram representing the reflectance PPG sensor and the ZenPPG instrumentation. Light emitting diodes in the sensor are switched ON/OFF by the multiplexer and current source. The current produced by the photodiode are converted into voltages by the transimpedance amplifier and time-demultiplexed. An external ADC digitises the analogue signals from the instrumentation before acquisition of by a personal laptop/computer in LabVIEW.

A reflectance PPG sensor was used to record signals from the forehead. The sensor comprises two red LEDs (KP-2012SRC-PRV, Kingbright, Taiwan) shining light at 660 nm, and two LEDs (KP-2012SF4C, Kingbright, Taiwan) emitting infrared light at 880 nm. At the centre of the PPG sensor, a silicon photodiode (TEMD5010X01, Vishay Intertechnology Inc., Malvern, PA, USA) detects the reflected light from the tissue. To protect the optical components, facilitate the attachment on the skin and cover from ambient light, the sensor features a circular-shape mechanical structure (Figure 1). In this study, two identical reflectance PPG sensors were used simultaneously to acquire PPG signals from two different locations on the forehead.

A research photoplethysmographic system (ZenPPG) was used to acquire PPG signals from the forehead. The system has been developed by the Research Centre for Biomedical Engineering at City, University of London and it allows acquiring raw PPG signals (AC + DC). Through microcontroller processing, the ZenPPG drives the PPG sensor by controlling the ON/OFF switching sequence of the LEDs (multiplexing) and their respective light intensities. The system comprises of transimpedance amplifiers to convert the light-proportional currents produced by the photodiode. By time-demultiplexing and sample-and-hold, the ZenPPG separates the mixed signals from the photodiode into red and infrared PPG signals. The system is able to simultaneously collect dual-wavelength PPG measurements from two independent channels and it can drive both custom-made and commercial PPG/pulse oximetry sensors [15]. An external Data Acquisition Card (USB-6212, National Instruments, Austin, TX, USA) digitises the analogue signals for on-line acquisition through a personal computer. Figure 1 shows a flow diagram with the main components of the PPG acquisition system used in this study.

A Near Infrared Spectroscopy monitor (NIRO 200NX, Hamamatsu Photonics, Japan) was used as a reference for the measures of the changes in oxygenated (ΔHbO${}_{2}$), reduced (ΔHHb), and total haemoglobin (ΔtHb). The monitor measures the changes in haemoglobin concentrations by shining light at three wavelengths (735, 810, and 850 nm) and by using the modified Beer-Lambert law to obtain ΔHbO${}_{2}$, ΔHHb, and ΔtHb from the light attenuations [16]. The NIRO 200NX can also quantify the tissue oxygenation index (TOI) by application of the Spatially Resolved Spectroscopy [17], but this parameter has not been used in this study.

### 2.2. Subjects, Measurement Set-Up and Protocol

Sixteen (16) healthy subjects, 13 males and 3 females, with a mean age of 28 ± 6.7 were recruited in the study. Ethical approval was gained from the Senate Research Ethics Committee at City, University of London and written consent was sought from all the volunteers prior to the commencing of the study.

**Figure 2:**
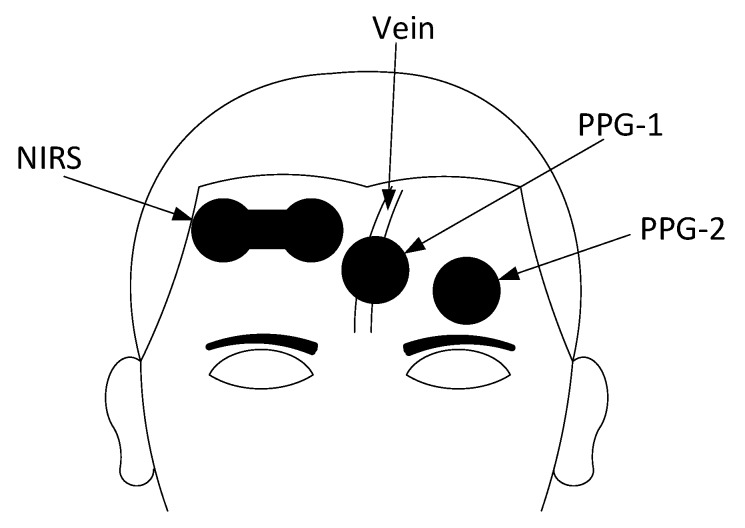
Positioning of the PPG and NIRS sensors on the forehead. One PPG sensor (PPG-1) was placed on top of the supratrochlear vein, while a second sensor (PPG-2) was placed on a vein-free area just above the eyebrow. The NIRS sensor was attached opposite with respect to the PPG-2 sensor.

Figure 2 shows the measurement set-up and the position of the sensors on the forehead. Two PPG sensors were attached on the forehead of each subject, with one PPG sensor (PPG-1) placed on top of a large vein and the second sensor (PPG-2) positioned on a region free of large superficial vessels. The supratrochlear vein, which lays approximately on the forehead’s midline, is one of the largest on the forehead and its location was marked on each volunteer prior to the attachment of the sensors. To expose the supratrochlear vein, each subject was slightly tilted from a supine position to approximately 10–15${}^{\circ }$ prior to sensors’ placement. The venous engorgement caused the extension and consequent exposure of the supratrochlear vein, allowing the marking of the sensor’s position. Similarly, the regions of the forehead free of large superficial vessels were marked as well. The region above the eyebrows was selected for the positioning of the PPG-2 sensor, since large vasculature is absent in this region, thus providing good PPG and pulse oximetry measurements [2]. Once the position of the supratrochlear vein and the other regions were marked, the tilting was stopped and the subject returned to a sitting position. After marking of the regions of interest on the forehead and allowing for recovery, the sensors were attached on the forehead by means of clear double-sided medical adhesives. The PPG-1 sensor was attached on top of the supratrochlear vein, while the PPG-2 was attached above the eyebrows. A NIRS sensor, with an emitter-detector distance of 4 cm, was attached on the opposite side with respect to the PPG-2 sensor and as high as possible, in order to avoid sinus cavities.

The protocol consisted of a negative tilting of the participants. The subjects were laying down supine on an electrically-controlled reclining bed and they were secured to the bed by apposite belts around their legs. After five minutes of baseline measurements, the bed was inclined at −30${}^{\circ }$ from supine (head-down). The subjects were kept in the tilted position for five minutes before restoring the baseline position (supine) for the final five minutes.

### 2.3. Data Analysis and Statistics

The analogue PPG signals from the instrumentation unit were digitised by a 16-bit ADC (USB-6212, National Instruments, USA) at a sampling frequency of 1 kHz. The digitised signals were acquired on a software application developed in LabVIEW (National Instruments, USA) for the acquiring, displaying and saving of the signals on real time. The signals from the NIRS monitor were also digitised at a sampling frequency of 1 kHz through a second ADC (PCIe 6321, National Instruments, USA) and acquired simultaneously to the PPG signals into the same software application.

The raw PPG signals (AC + DC) acquired from the forehead were split into the respective components by digital filters. The AC PPG component was obtained by band-pass filtering the raw signals between cut-off frequencies of 0.5–7 Hz (zero-phase IIR Butterworth filter, 6th order). The DC PPG component was separated by a low-pass filter at a cut-off frequency of 0.1 Hz (zero-phase IIR Butterworth filter, 6th order) [18]. All signals were downsampled to 100 Hz prior to filtering.

Changes in oxygenated, reduced and total haemoglobin can be estimated from changes in light attenuations by applying the differential approach of the modified Beer-Lambert law [13]. Assuming that oxygenated (ΔHbO${}_{2}$) and reduced haemoglobin (ΔHHb) are the only chromophores changing concentration in the sampled volume throughout the measurement, changes in light attenuations at two wavelengths can be related to ΔHbO${}_{2}$ and ΔHHb through the set of simultaneous equations in Equation (1).

(1) $$\Delta \Phi_{R}=\left(\alpha_{{{\mathrm{HbO}}_{2}}_{R}}\cdot \Delta {\mathrm{HbO}}_{2}+\alpha_{{\mathrm{HHb}}_{R}}\cdot \Delta \mathrm{HHb}\right)\cdot d\cdot \mathrm{DPF}+\Delta G\;\;\Delta \Phi_{\mathrm{IR}}=\left(\alpha_{{{\mathrm{HbO}}_{2}}_{\mathrm{IR}}}\cdot \Delta {\mathrm{HbO}}_{2}+\alpha_{{\mathrm{HHb}}_{\mathrm{IR}}}\cdot \Delta \mathrm{HHb}\right)\cdot d\cdot \mathrm{DPF}+\Delta G$$

where ΔΦ${}_{R}$ and ΔΦ${}_{IR}$ are the light attenuations at red and infrared respectively, $\alpha_{{{\mathrm{HbO}}_{2}}_{R}}$ and $\alpha_{{\mathrm{HHb}}_{R}}$ are the extinction coefficients of oxygenated and deoxygenated haemoglobin at the red wavelength (660 nm), $\alpha_{{{\mathrm{HbO}}_{2}}_{IR}}$ and $\alpha_{{\mathrm{HHb}}_{IR}}$ are respectively the extinction coefficients of oxygenated and deoxygenated haemoglobin at the infrared wavelength (880 nm), d is the interoptode distance, DPF is the differential pathlenght factor and G is a coefficient indicating scattering in the tissue.

The term G in Equation (1) can be neglected assuming that scattering remains constant during the measurements (ΔG = 0) and solving the set of equations in Equation (1) for ΔHbO${}_{2}$ and ΔHHb yields to the results in Equations (2) and (3).

(2) $$\Delta {\mathrm{HbO}}_{2}\;=\;\frac{\Delta \Phi_{R}\cdot \alpha_{IR_{\mathrm{HHb}}}-\Delta \Phi_{IR}\cdot \alpha_{R_{\mathrm{HHb}}}}{\alpha_{R_{{\mathrm{HbO}}_{2}}}\cdot \alpha_{IR_{\mathrm{HHb}}}-\alpha_{IR_{{\mathrm{HbO}}_{2}}}\cdot \alpha_{R_{\mathrm{HHb}}}}$$

(3) $$\Delta \mathrm{HHb}\;=\;\frac{\Delta \Phi_{IR}\cdot \alpha_{R_{{\mathrm{HbO}}_{2}}}-\Delta \Phi_{R}\cdot \alpha_{IR_{{\mathrm{HbO}}_{2}}}}{\alpha_{R_{{\mathrm{HbO}}_{2}}}\cdot \alpha_{IR_{\mathrm{HHb}}}-\alpha_{IR_{{\mathrm{HbO}}_{2}}}\cdot \alpha_{R_{\mathrm{HHb}}}}$$

Since the optical pathlength travelled by the light (expressed by the term d · DPF in Equation (1)) is unknown for this kind of PPG measurements, the solutions in Equations (2) and (3) represent the changes in ΔHbO${}_{2}$ and ΔHHb relative to the unknown optical pathlength (concentration × optical pathlength), expressed in mM·cm.

The light attenuations changes ΔΦ${}_{R}$ and ΔΦ${}_{IR}$ in Equations (2) and (3) can be derived from the DC PPG signals by applying Equations (4) and (5).

(4) $$\Delta \Phi_{R}=\ln\left(\frac{DC_{R}\left(0\right)}{DC_{R}\left(t\right)}\right)$$

(5) $$\Delta \Phi_{IR}=\ln\left(\frac{DC_{IR}\left(0\right)}{DC_{IR}\left(t\right)}\right)$$

where $DC_{R}\left(0\right)$ and $DC_{IR}\left(0\right)$ are respectively the red and infrared DC PPGs at the start of the measurement (baseline), whereas $DC_{R}\left(t\right)$ and $DC_{IR}\left(t\right)$ are respectively the red and infrared DC PPG throughout the measurement.

The changes of the variables in the population were expressed by estimating the median and the interquartile range (IQR = Q${}_{3}$ − Q${}_{1}$) calculated over the central three minutes of each protocol step (baseline, tilting and recovery).

Statistically significant changes were assessed by pairwise comparisons between baseline and interventions (i.e., baseline-tilting and baseline-recovery) by using a non-parametric test (Wilcoxon signed rank test) [19]. A p-value p < 0.05 was considered statistically significant.

Trending comparison was performed between the PPG and NIRS measurements to assess the reliability of the PPG in measuring changes in ΔHbO${}_{2}$, ΔHHb and ΔtHb. Trends (or direction of the changes) in the haemoglobin concentrations from PPG were assessed by calculating the correlation with NIRS measures and by assessing trending agreement. The correlation was evaluated by estimating the mean Pearson’s correlation coefficients *r* and the 95% confidence intervals (CI) for the population investigated. The trending agreement was evaluated by four-quadrant plot analysis [20]. The concordance rate *C* was calculated as the ratio of the number of points in the first and third quadrant and the total number of points in all four quadrants. An exclusion zone of 15% was used for the calculations in order to avoid the ’central zone effect’ [20]. A concordance rate C ≥ 90% was considered satisfactory for indicating good trending agreement. All the signal processing and statistical analysis were performed in Matlab (Mathworks, Natick, MA, USA).

## 3. Results

### 3.1. Haemoglobin Concentration Changes from PPG

**Figure 3:**
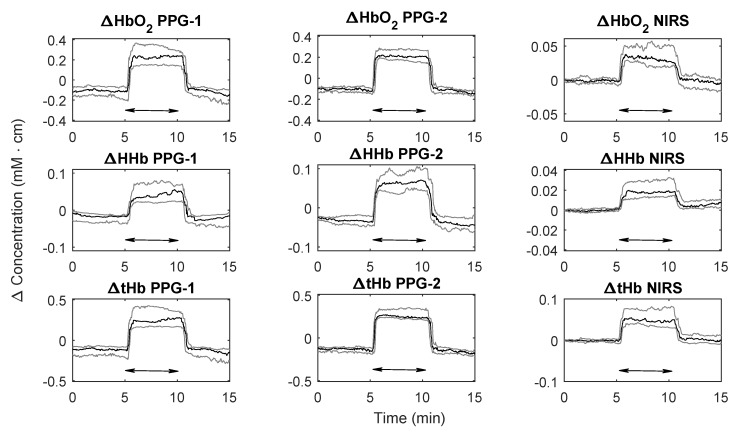
Changes in ΔHbO${}_{2}$, ΔHHb, and ΔtHb estimated from the PPG sensors and the reference NIRS monitor during the bed tilting protocol. Black lines indicate the medians across all subjects, while the grey lines show the upper and lower quartiles among the subjects. The double arrows indicate the duration of the head-down tilt.

Figure 3 shows the changes in ΔHbO${}_{2}$, ΔHHb, and ΔtHb measured from both PPG sensors and the simultaneous NIRS measurements during the bed tilting protocol. The negative tilting caused a concomitant increase in both ΔHbO${}_{2}$ and ΔHHb. In particular, the rise in ΔHbO${}_{2}$ was caused by the increased arterial blood flow caused by gravity. Similarly, the change in ΔHHb resulted from the venous engorgement in the upper part of the body caused by the impaired return of venous blood during tilting. Total haemoglobin, which represents the total blood volume underneath the sensor, increased as well during tilting. In a similar fashion as the PPG-derived parameters, the changes in ΔHbO${}_{2}$, ΔHHb, and ΔtHb from the NIRS monitor changed accordingly during the tilting.

**Figure 4:**
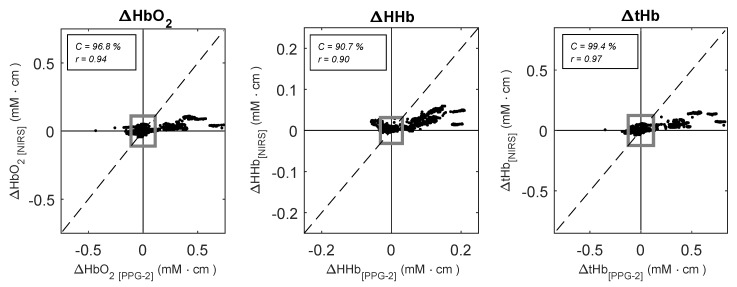
Four-quadrant plots analysis between ΔHbO${}_{2}$, ΔHHb, and ΔtHb estimated by NIRS (y-axis) and the PPG-2 sensor (x-axis) during the tilting protocol. Dotted lines represent the line of identity. A total number of n = 100 pair points for each subject were used for the plots. The grey square indicates the 15% exclusion zone.

The changes in ΔHbO${}_{2}$, ΔHHb, and ΔtHb measured by the PPG-2 sensor and the NIRS monitor during the protocol are summarised in Table 1. Tilting caused significant changes in all the haemoglobin species measured by PPGs and NIRS (p < 0.001). As it can also be seen from Figure 3, the changes measured by PPG-2 followed the same trends of the NIRS, with correlation coefficients *r*(95% CI) equal to *r* = 0.94 (0.83, 0.98), *r* = 0.90 (0.73, 0.96), and *r* = 0.97 (0.91, 0.99) for ΔHbO${}_{2}$, ΔHHb, and ΔtHb respectively. Trending was also assessed by four-quadrant plot analysis, which provides an evaluation of directionality of change between two measurement techniques. The results of the four-quadrant plot analysis between the PPG-2 sensor and NIRS for ΔHbO${}_{2}$, ΔHHb, and ΔtHb is illustrated in Figure 4. The analysis yielded concordance values of C = 96.8%, C = 90.7%, and C = 99.4% for ΔHbO${}_{2}$, ΔHHb, and ΔtHb respectively.

### 3.2. Contribution of a Large Vein to the Haemoglobin Concentration Changes from PPG

As shown in Figure 3, the PPG sensor placed on top of the large vein (PPG-1) indicated the same changes in the parameters as the PPG sensor positioned distant from the large vasculature (PPG-2) and as the reference NIRS measurements.

**Figure 5:**
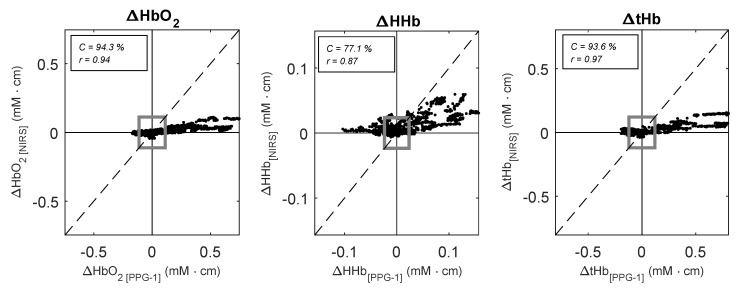
Four-quadrant plots analysis between ΔHbO${}_{2}$, ΔHHb, and ΔtHb estimated by NIRS (y-axis) and the PPG-1 sensor (x-axis) during the tilting protocol. Dotted lines represent the line of identity. A total number of n = 100 pair points for each subject were used for the plots. The grey square indicates the 15% exclusion zone.

When compared to the reference NIRS monitor, the sensor on the vein correlated well with NIRS measurements, with correlation coefficients r = 0.94 (95% CI: 0.85, 0.98), r = 0.87 (95% CI: 0.66, 0.95), and r = 0.97 (95% CI: 0.91, 0.99), for ΔHbO${}_{2}$, ΔHHb and ΔtHb respectively. However, the four-quadrant plot analysis showed in Figure 5 revealed a poor concordance between the changes in ΔHHb measured from the PPG-1 sensor and the NIRS monitor (C = 77.1%), while ΔHbO${}_{2}$ and ΔtHb showed a good concordance of C = 94.3% and C = 93.6% respectively.

**Figure 6:**
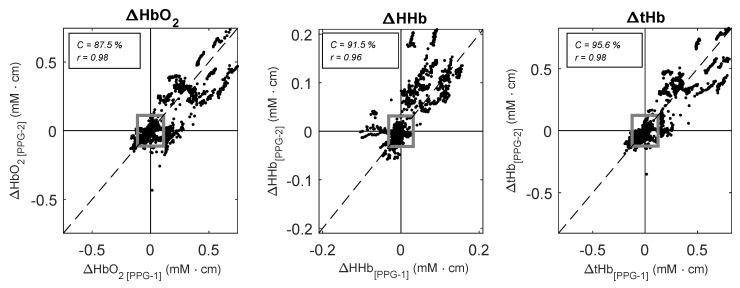
Four-quadrant plots analysis between ΔHbO${}_{2}$, ΔHHb, and ΔtHb estimated by the PPG-2 (y-axis) and the PPG-1 sensor (x-axis) during the tilting protocol. Dotted lines represent the line of identity. A total number of = 100 pair points for each subject were used for the plots. The grey square indicates the 15% exclusion zone.

The sensor placed on top of the large vein followed the same trends as the identical PPG sensor positioned on the vein-free area above the eyebrow. There was a good correlation between the variables measured by the two PPG sensors, with correlation coefficients r = 0.98 (95% CI: 0.94, 0.99), r = 0.96 (95% CI: 0.89, 0.98), and r = 0.98 (95% CI: 0.96, 0.99), for ΔHbO${}_{2}$, ΔHHb and ΔtHb respectively. Four-quadrant plot analysis illustrated in Figure 6 yielded concordance values of C = 87.5%, C = 91.5%, and C = 95.6% for ΔHbO${}_{2}$, ΔHHb, and ΔtHb respectively.

**Figure 7:**
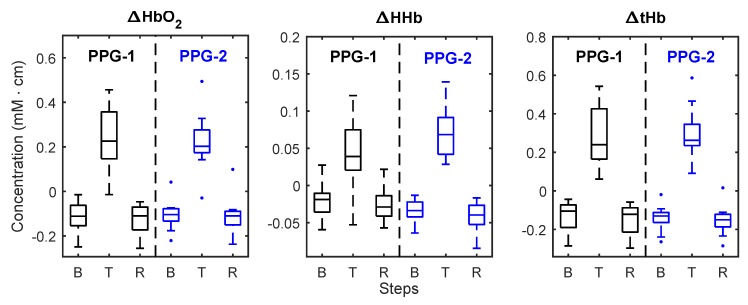
Box and whiskers plot of the mean changes in ΔHbO${}_{2}$, ΔHHb, and ΔtHb measured by the two PPG sensors during baseline (B), tilting (T) and recovery (R). PPG-1: sensor positioned on top of the large vein; PPG-2: sensor placed on the vein-free area above the eyebrow.

Differences between the two PPG measurement locations were assessed by testing for statistical significance between the means averaged over the central three minutes of each stage (i.e., baseline, tilting, and recovery). Figure 7 shows the box and whiskers plots of the means of haemoglobin concentrations calculated from both PPG sensors. When compared to the sensor placed distant from large vessels, the changes in reduced haemoglobin ΔHHb estimated from the PPG sensor on the large vein showed a significant difference during baseline (p = 0.03), tilting (p = 0.03), and recovery (p = 0.01). Changes in oxygenated haemoglobin (ΔHbO${}_{2}$) and total haemoglobin (ΔtHb) estimated from the sensor on the vein did not show any significant difference (p > 0.05) compared to the sensor placed away from any vein.

Mean absolute errors (MAE) were calculated between the haemoglobin concentrations estimated from the two PPG sensors. The MAE values were calculated in the three minutes central section of each stage (i.e., baseline, tilting, and recovery) and are summarised in Table 2. MAE analysis revealed a larger error in reduced haemoglobin compared to oxygenated and total haemoglobin.

## 4. Discussion

It is well documented that haemoglobin concentrations changes measured by NIRS can reveal changes in perfusion induced by changes in posture such as the Trendelenburg position [21,22,23]. Therefore, this study assessed whether PPG could be used to detect the same posture-dependent changes. Comparing ΔHbO${}_{2}$, ΔHHb and ΔtHb from a PPG sensor on the forehead with the same measurements derived from a reference NIRS sensor provides the means to validate the capability of the PPG to monitor these posture-induced changes. The results from this study indicated that ΔHbO${}_{2}$, ΔHHb and ΔtHb estimated from the PPG can be used to monitor the haemodynamic changes, such as venous pooling, caused by bed tilting. The high correlation and the good concordance agreement assessed by four-quadrant plot analysis indicated that the PPG-derived parameters trended well with the reference NIRS measurements. In this regard, the four-quadrant plot analysis proved to be an additional and useful tool that, to our knowledge, has never been used to assess the directionality of change between these two techniques. These results confirm the ability to derive the relative changes in haemoglobin concentrations from PPG signals. In our previous publications, we demonstrated that these measurements can be obtained from the forearm during different degrees of vascular occlusions [13,14], but this study gave the opportunity to further validate the use of this technique on PPG signals acquired from a more common location such as the forehead.

Measuring the relative changes in haemoglobin concentrations from PPG signals acquired from the forehead could be interesting since PPG measurements from this anatomical location can be affected significantly by the venous circulation. Although in normal conditions this contribution can be neglected, the venous pooling caused by the posture of the patient (e.g., Trendelenburg position) can increase the venous presence in the vascular bed under the sensor [4,12]. This causes morphological alterations in the signal and erroneous estimation of the arterial oxygen saturation in forehead pulse oximetry. Elastic headbands can reduce the manifestation of this problem [4] or qualitative assessments on the position of the patient and the signal morphology can reveal a suspected venous interference on the signals [3,9]. Considering that there are no quantitative methods to assess this, monitoring the relative changes in haemoglobin concentrations from the DC component of the same PPG signals may provide a useful tool to reveal the presence of venous blood in the volume sampled by the optical sensor. The main advantages of this method are its relative simplicity and the fact that it does not require any additional sensors or instrumentation since the parameters can be estimated from the same signals acquired for SpO${}_{2}$ measurements (i.e., red and infrared PPG signals).

Our method proposed in this paper relies on the spectroscopical separation of ΔHbO${}_{2}$, ΔHHb and ΔtHb from DC PPG signals by applying the modified Beer-Lambert law. The main challenge that remains is the removal/subtraction of the cardio-synchronous venous pulsations that appear in the PPG signals during venous pooling. It is hoped that the advancements in signal processing techniques in recent years could help in overcoming this issue. In this regard, features extraction algorithms such as the one developed by Yousefi and Nourani could be used to separate the time-domain signals into their arterial and venous component [24]. This approach could be useful in isolating the venous pulsations from the forehead signals in an attempt to improve the estimation of SpO${}_{2}$.

Previous work by Mannheimer demonstrated that a correct positioning of PPG/pulse oximetry sensors, avoiding large arteries on the forehead, improves the estimations of SpO${}_{2}$ [2]. Similarly, in this study, we wanted to investigate the influence that large veins on the forehead could have on the estimation of the relative changes in haemoglobin concentrations. When compared to an identical sensor placed in a vasculature-free area, the positioning of the sensor on top of a large vein caused a significant difference and a higher mean absolute error in the estimation of ΔHHb. Interestingly, the presence of a vein underneath the sensor caused an underestimation of ΔHHb and higher variability in this parameter. Also, placing the PPG sensor on top of a large vein on the forehead caused a decrease in the trending agreement of the PPG-derived ΔHHb when compared with the reference NIRS measurements. With marked changes and less variability in the measurements, it appears that the capillary bed (i.e., PPG-2) had a higher capability to show changes in ΔHHb compared to the same measurement from the top of a large vein (i.e., PPG-1). A possible reason for this behaviour may be caused by limited extensibility and capacity of the veins during venous pooling compared to the capillary bed. Another important aspect of this comparison is the optical heterogeneity caused by the introduction of a large vein in the sampled volume. Optical techniques such as pulse oximetry/PPG and NIRS rely on the homogeneity of the measurement area and the presence of a large vessel underneath the sensor can create regions in which photons could undergo abnormal absorption or scattering/reflection [2,25]. However, in this current study, it is difficult to determine the extent of these two effects (i.e., the physiological difference in reduced haemoglobin in the two areas or the optical heterogeneity), but it could be assumed that both contributed to the observed biases. In this regard, tailored optical simulations could help in identifying the contributions of these phenomena to the measurements.

As observed in both the four-quadrant plot analysis and the means of the changes, there were some quantitative differences between PPG and NIRS measurements. These differences could be mainly attributed to the different sampling volumes interrogated by the two instruments. NIRS can penetrate deep into the skull, reaching the cerebral surface [16], while the interrogation depth of the PPG is limited to the skin [26]. Other functional differences between the PPG and NIRS devices utilised in the study (i.e., wavelengths, algorithms, etc.) could have also played a role in these quantitative differences.

The main limitation of the current method employed to derive changes in haemoglobin concentrations from the PPG (i.e., modified Beer-Lambert law) is the inability to determine absolute values of haemoglobin concentrations, thus only expressing the relative changes from an arbitrary baseline. This is a well-known limitation of continuous-wave instruments (such the one used in this study) due to their incapability of measuring the optical pathlengths and the absolute light intensities [25]. Although using a time or phase domain instrument could overcome this issue, the purpose of this study was to use standard pulse-oximetry/PPG technology for estimating the changes in ΔHbO${}_{2}$, ΔHHb and ΔtHb during a posture change.

## 5. Conclusions

This paper investigated the feasibility of using PPG signals from the forehead to estimate relative changes in haemoglobin concentrations during a head-down tilting protocol on healthy volunteers. The promising comparative results with reference NIRS measurements on the forehead indicate that the method could be used as a tool to measure venous pooling under the sensor and identify situations in which an increase in venous blood could degrade the forehead PPG signals and cause erroneous SpO${}_{2}$ readings. However, the positioning of the PPG sensor on the forehead plays an important role in the accuracy of the results. Avoiding large vasculature present on the forehead improves the trending capabilities of the PPG as well as reducing biases and variabilities in the measurements.

## Figures and Tables

**Table 1:** Median changes (IQR = Q${}_{3}$ − Q${}_{1}$) of relative haemoglobin concentrations changes estimated by PPG and NIRS during baseline, tilting, and recovery. The PPG-derived haemoglobin concentrations refer to the PPG-2 sensor, which was placed above the eyebrow. The values are averaged over the central three minutes of baseline, tilting, and recovery. Values in mM·cm.

	Baseline	Tilting	Recovery
	(mM·cm)	(mM·cm)	(mM·cm)
ΔHbO${}_{2}$ (PPG)	−0.104 (0.056)	0.202 (0.102)	−0.110 (0.061)
ΔHHb (PPG)	−0.033 (0.020)	0.068 (0.049)	−0.039(0.026)
ΔtHb (PPG)	−0.130 (0.052)	0.262 (0.111)	−0.150 (0.065)
ΔHbO${}_{2}$ (NIRS)	−0.001 (0.005)	0.032 (0.030)	0.001 (0.017)
ΔHHb (NIRS)	0.000 (0.003)	0.018 (0.016)	0.004 (0.008)
ΔtHb (NIRS)	−0.001 (0.005)	0.050 (0.041)	0.004 (0.018)

* Significant change from baseline (p < 0.001).

**Table 2:** Mean absolute errors (MAE) between haemoglobin concentrations estimated from the two PPG sensors during the bed tilting protocol.

	Baseline	Tilting	Recovery
	(mM·cm)	(mM·cm)	(mM·cm)
ΔHbO${}_{2}$	−0.007	0.014	−0.011
ΔHHb	0.013	−0.026	0.013
ΔtHb	0.005	−0.011	0.002
